# The Potential of Polyelectrolyte Multilayer Films as Drug Delivery Materials

**DOI:** 10.3390/ijms23073496

**Published:** 2022-03-23

**Authors:** Joanna Potaś, Katarzyna Winnicka

**Affiliations:** Department of Pharmaceutical Technology, Medical University of Białystok, Mickiewicza 2c, 15-222 Białystok, Poland; joanna.potas@umb.edu.pl

**Keywords:** polyelectrolyte complex, polyelectrolyte multilayer film, drug delivery material, multifunctional polymeric material

## Abstract

Polyelectrolyte multilayers (PEMs) represent a group of polyelectrolyte complex (PEC)–based materials widely investigated in the biomedical and pharmaceutical sciences. Despite the unflagging popularity of the aforementioned systems in tissue engineering, only a few updated scientific reports concerning PEM potential in drug administration can be found. In fact, PEM coatings are currently recognized as important tools for functionalizing implantable scaffolds; however, only a small amount of attention has been given to PEMs as drug delivery materials. Scientific reports on PEMs reveal two dominant reasons for the limited usability of multilayers in pharmaceutical technology: complex and expensive preparation techniques as well as high sensitivity of interacting polyelectrolytes to the varieties of internal and external factors. The aim of this work was to analyze the latest approaches, concerning the potential of PEMs in pharmacy, chemical technology, and (primarily) tissue engineering, with special attention given to possible polymer combinations, technological parameters, and physicochemical characteristics, such as hydrophilicity, adhesive and swelling properties, and internal/external structures of the systems formed. Careful recognition of the above factors is crucial in the development of PEM-based drug delivery materials.

## 1. Introduction

**Figure 1:**
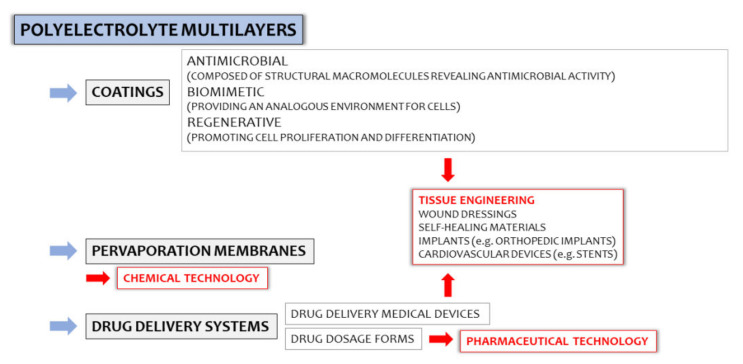
The applicability of polyelectrolyte multilayers (PEMs) in the biomedical and pharmaceutical fields.

Polyelectrolyte multilayers (PEMs) are a group of polyelectrolyte complex (PEC)-based materials, in which usability in pharmaceutical and chemical technologies, the food industry, as well as in tissue engineering have been investigated throughout the years (Figure 1). Despite high interest in the development of PEM-based drug delivery systems in the early 2000s, a lack of updated scientific reports concerning the potential of multilayer systems in drug administration was observed. In fact, PEM coatings are currently recognized as important tools for functionalized particulate systems [1]; however, little attention has been given to multilayer films as potential drug carriers. It can be assumed that PEM preparation techniques highly affect their limited usability in the pharmaceutical industry since they are mostly based on complex and expensive devices (e.g., automatic dipping machines, inkjet printers, or laboratory spinnerets), and drug entrapment efficiency is usually very low. Considering the specificity of PECs as structures “shaped” by various external and internal factors (pH, ionic strength, polyelectrolytes characteristics, e.g., molecular weight, charge density, viscosity), the need for careful optimization of the technological process might be another obstacle in the widespread use of PEC-based multilayer films.

Keeping in mind the potential of PEMs and the possibilities that such multifunctional, complex materials might offer (i.e., in terms of novel, targeted, systems development), we delved deeper into the technological and applicability aspects of PEMs. Better insight into the mechanisms of PEM formation and the factors influencing that process are crucial to better understand the usability of these complex systems.

### 1.1. PECs

PECs are three-dimensional structures created by oppositely charged polyelectrolytes (both polymers and active substances) combined by reversible electrostatic interactions, additionally supported by hydrogen and/or hydrophobic forces [2]. Positively and negatively charged macromolecules (polycations and polyanions, respectively) are recognized in the variety of polyelectrolytes that interact with each other. While chitosan and its derivatives with cationic amine groups are the most commonly used polycations, a plethora of polyanions, including natural, semi-synthetic and synthetic, have been investigated with regard to PEC formation. They are usually the carriers of carboxylic or, less frequently, sulfuric acid groups that electrostatically interact with chitosan.

Among the numerous classification criteria of PECs, the one based on the structural model of polycomplexes in water seems to be particularly helpful when it comes to further analyses. According to the above, PECs can be divided into stoichiometric and non-stoichiometric types. Non-stoichiometric PECs are composed of polyelectrolytes with significant differences in their molecular weights and charge densities, leading to partial polymer–polymer interactions and an excess of either positively- or negatively-charged functional groups responsible for water solubility. In turn, insoluble stoichiometric PECs are characterized by the equivalent ratio of charges, fully interacting with each other [2]. The majority of designed PEC-based drug delivery systems and biomedical devices “focus” on the attempts of chitosan complexation by using different polyanions [3,4,5]. These attempts are made to develop novel drug delivery systems with unique properties as compared with single interacting polymers, which improve the stability and physicochemical performances of chitosan, while simultaneously maintaining its multifunctional character (e.g., antibacterial, antifungal, anti–inflammatory, or healing activities).

PEC preparation does not require the addition of toxic crosslinking agents; however, usually takes place in an aqueous environment after mixing polyelectrolytes. Nevertheless, rapid interactions between oppositely charged polymers usually result in PEC coacervation or precipitation, which affect the homogeneity of the polymer mixtures and their further utilization. Separation of insoluble PEC particles from transparent solutions is one of the applied method that leads to a homogeneous system [6]. From the other side, optimization of the interpolymer complexation process is highlighted as the key to tailor PEC characteristics and prevent unwanted precipitation. Considering the number of factors influencing PECs, including polymer characterization (e.g., molecular weight, charge distribution, pKa, solubility, chain flexibility), ionic strength, pH values that affect the degree of ionization of the polyelectrolytes, temperature, concentrations of the interacting components, polymer ratio, as well as the preparation technique by itself—a detailed analysis of the polymers prior to the highly controlled process of PEC formation is always required [2,7,8].

### 1.2. Potential of PEMs

PEMs are layered materials composed of consecutively deposited layers of oppositely charged polyelectrolytes and interfacial layers of ionically-interacting polymeric chains creating PECs. Depending on the applied preparation technique, each polyelectrolyte layer might be deposited automatically via an inter alia dipping, spinning, or spraying technique, or by simple “casting” of the individual polymer solutions on Petri dishes (or other similar containers). PEMs are generally identified with automatically formed materials, where layer-by-layer (Lbl) assembly results in alternate depositions of molecularly thick polyanion and polycation layers, with nanoscale control over the composition, structure, size, and shape of the systems formed [9]. Among the different variants of Lbl techniques developed in the early 1990s, the dipping coating method was the most popular. It is basically composed of three repetitive steps, beginning with the adsorption of polyelectrolytes onto a hard substrate (e.g., glass, silicon, polycarbonate, polyketone); after, it is dipped in a polyelectrolyte solution with subsequent slow rearrangement of polymer molecules within the layers [10]. Then, the excess polymer is removed by washing the substrate with an adequate medium; the above procedure can be repeated for the oppositely charged component of the film. The substrate can either remain as a composite of the final material or be removed by using physical and chemical treatments. Depending on the necessity, the varieties of the charged substrates, including large scale and nanoscale surfaces, can be coated with PEMs. In parallel, less or more complex multilayer materials can be obtained. The application of protein-supported substrates in the technology of PEMs, formed from poly(diallyldimethylammonium chloride) and poly(sodium styrene sulfonate), has recently been verified [11]. The authors concluded that pre-adsorbed layers of β–lactoglobulin might increase the elasticity of the solid substrates with simultaneously more regular growth of the single polyelectrolyte layers. For drug-loaded PEMs, an additional step that relies on dipping the substrate with a build-up film in a drug solution can be indicated. Since incorporation of a drug into Lbl self-assembling materials relies on time-limited adsorption and penetration of a dissolved substance into a polymeric matrix, constraints of the method with regard to poorly soluble or unstable substances (e.g., macromolecules) should be underlined. Expensive and complex technologies, as well as obstacles in large-scale applications of the dipping-based Lbl technique, are equally important reasons for its limited use in the biomedical field [12].

Regarding the above limitations, films developed via the spin coating technique (which relies on casting a solution onto either a spinning or stationary substrate, being subsequently spun) was explored by Bodurov et al. [10]. It has been proven that more homogenous films might be produced by using the aforementioned technique as compared to the dipping coating procedure since the centrifugal and viscous forces, as well as air shear, are responsible for elimination of weakly bound polyelectrolytes and film dehydration [10,13]. In the study by Bodurov et al., low molecular weight chitosan and xanthan gum acetate buffer solutions, with a pH of 4.5 being optimal for ionization of both polyelectrolytes (pKa of chitosan is 6.5 and xanthan gum–3.1), and ionic strength of 0.1 M, were alternately deposited on the negatively charged poly(DL–lactide) substrate by using the dipping and spinning method. The weight ratio of polycation to polyanion in the neighboring layers was 2:1, as a result of the two-fold more concentrated chitosan solution preparation compared to xanthan gum dispersion (0.1% (*w*/*v*) vs. 0.05%, respectively). Film thickness measurements, which were performed by using a surface refractive index analysis, showed noticeably faster growth of dipping-assembled materials compared to spinning-assembled materials. As a result of the spinning procedure, less intense diffusions and interpenetrations of the “uncomplexed” polyelectrolytes inside the film matrices were observed, which affected preparation of the significantly thinner composites. We should note that, for the Lbl technique based on spray depositing, the rinsing step might be omitted, with a faster build-up process as a result [12].

Apart from the aforementioned Lbl techniques, we also focus on the (widely known in the pharmaceutical technology) solvent evaporation method and its use in the development of—admittedly less complex, but more affordable and easier to scale-up—PEMs. In this technique, single polyelectrolyte solutions are casted alternately on a Petri dish, although the next layer can be casted only after a preliminary gelation of the previous one. For that purpose, an adequately long drying procedure prior to the next layer deposition should be included in the technological process.

In addition to the confirmed usability of Lbl-assembled composites in photo–electrochemistry or, e.g., anti-corrosion coating preparations, the potential of PEMs in developing antimicrobial, regenerative, or biomimetic coatings, as well as functionalized medical devices, cannot be overlooked. Significant attention is given to PEMs as potential biomimetic materials due to the possibilities of the Lbl technique, i.e., in nanoscale control over physical and chemical processes involved in the biomaterials designing. Detailed characteristics of PEM applicability in materials engineering was the topic of many (recently published) review articles [14,15,16], but with only cursory evaluations of PEMs as potential drug dosage forms. Considering the problem of PEC coacervation or precipitation highly affecting polycomplex-based systems uniformity, Lbl deposition, either by dipping coating or the solvent evaporation technique, reduces the contact between oppositely charged macromolecules; thus, the risk of preparation of inhomogeneous systems with poor mechanical characteristics can be eliminated. Accordingly, a wider spectrum of polycations and polyanions might be easily combined. Considering the multifunctional characters of many pharmaceutically applied polymers, the idea of designing highly optimized multicomponent drug delivery platforms is worth a discussion.

### 1.3. Physicochemical Background of PEM Formation

It cannot be overlooked that, by incorporating different entities in multilayer materials, structures with novel physicochemical behaviors are created. This observation is common for all PEC composites characterized by unique properties arising from combining different polymers. Nevertheless, the similarity of polycomplexes and PEMs does not only concern their novelty; high susceptibility of the structures to the variety of internal and external stimuli should also be highlighted. As Das et al., emphasized [9], the only way to wider usability of PEMs is via better recognition of the kinetics and transport phenomena responsible for their formations as well as understanding the factors affecting their performance.

It is commonly known that PEC formation is a favorable process since it is associated with energy decline, being the result of an interruption of an electrical double layer surrounding the polyelectrolyte molecules, counterion release responsible for the entropy growth [9,17], and it is driven by the loss of water molecules [18]. Accordingly, polymer association underlying the process of PEM formation can occur [19]. The majority of counterions are removed at the stage of the rinsing process relevant to the Lbl preparation technique, which, according to the above, should encourage a polycation–polyanion complexation. It should be noted that high ionic strength accompanying PEC formation determines only partial neutralization of the charge, increased molecular mobility, and soluble polycomplexes synthesis [9]. Furthermore, according to the observations made by Tsuchida et al. [20], partially complexed polyelectrolytes seem to be more eager to react with oppositely charged polymeric chains than non-electrostatically bound polyelectrolytes. High molecular weight polymers are regarded as more easily adsorbed on a substrate with relatively irreversible bond formation as compared to those with low molecular weight. As previously described, precipitated, soluble, colloidal suspended PECs, or dense coacervates might be formed during Lbl polymer deposition, depending on the applied parameters. For example, upon increasing polymer concentration or chain length, the probability of polycomplexes coacervation grows [21]. Generally, it is assumed that soluble PEC formation results in no PEMs or thin layers susceptible to erosion as compared to stoichiometrically insoluble structures, creating thick and rapidly growing materials in a linear way [22]. In the linear mode of film growth, polyelectrolytes only interact with their neighboring oppositely charged layers. In contrary, exponential growth is relevant to polyelectrolytes with low molecular weight and charge density, enhanced flexibility, and hydrophilicity of the polymeric chains. Multilayer systems obtained in that way might be characterized as gel-like materials with loosely packed macromolecules interacting with each other over the entire thickness of the film [23].

**Figure 2:**
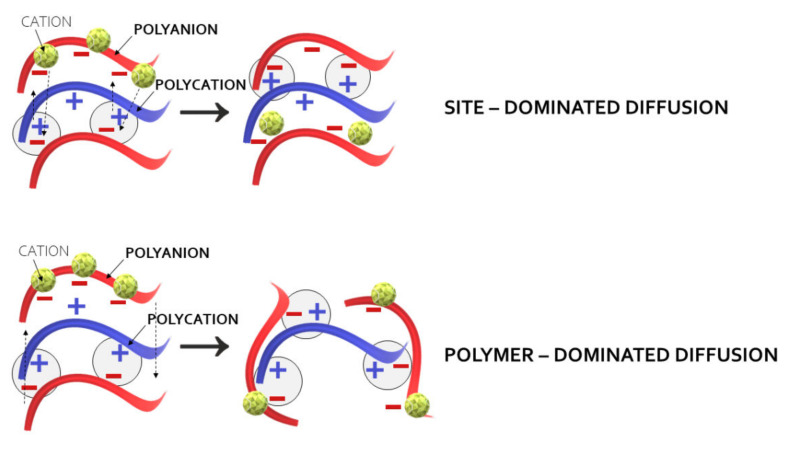
Site- and polymer-dominated diffusion mechanisms of PEM formation.

There are different factors, such as pH, ionic strength, or temperature, which determine the mechanisms of PEM formation. It has been observed that linear growth modes are based on the exothermic processes while the exponential growth of the films is associated with endothermic complexation [12]. The outcomes, published by Schlenoff et al., and Fares et al. [18,24], greatly contributed, in regard to better recognition of mechanisms of PEM formation. Based on the sensitive isotopic labeling techniques, the authors indicated the importance of the site diffusion mechanism in Lbl polyelectrolyte depositions. Rather than relying on interdiffusion of polymer molecules throughout the entire film in PEM fabrication, they emphasized the role of changes within its extrinsic sites composed of polyelectrolyte repeat units balanced by counterions. In fact, diffusion coefficients noted for polymers themselves were significantly lower than those calculated for PEM extrinsic sites. As presented in Figure 2, the reorganization of the PEC network after contact with a new polymeric layer promotes ionic interactions between interfacial surfaces by “flipping” around the charged functional groups in the site diffusion mechanism. In turn, the theory of polymer-dominated diffusion bases, on the concept of a polymeric chain movement throughout the PEC network as the new film layer, was applied.

Although PECs might be composed of many oppositely charged polymers, compositions of PEMs are typically limited to one polycation and one polyanion that perform maximum affinity to electrostatic interactions. To provide better insight into physicochemical behavior and possible utilization of PEMs, a short characterization of the polyelectrolytes used in the referenced studies is included in Table 1, with special attention given to their biomedical potentials. Apart from the typical polyelectrolytes giving structure to the carrier, charged active substances, such as heparin or tannic acid, were also listed as possible PEM components.

## 2. Applicability of PEMs

### 2.1. PEMs as Drug Delivery Systems

In this section, we review the utility of PEMs in drug carrier development, considering the beneficial application properties of pharmaceutical films, as being easy to administer, microbiologically stable, and versatile for either local or systemic use dosage forms. The majority of the recalled scientific reports concern the utilization of PEM coatings in the biofunctionalization of medical devices intended for delivery of various active substances (Table 2) [29]. In fact, expensive and complex technology, as well as obstacles in large scale applications of the sophisticated Lbl techniques, are listed as the main reasons for the limited use of PEMs for strictly pharmaceutical purposes. Furthermore, leakage of low molecular weight drugs before the intended degradation, and providing controlled release, are still challenging in the fabrication of PEM-based drug carriers [14,30]. Accordingly, different approaches proceeded in order to improve drug entrapment efficiency.

As previously mentioned, simple PEC films obtained via “easy to scale-up” and affordable solvent evaporation techniques could be good alternatives for drug-loaded system preparations, where, apart from the issues related to the polymer–polymer interactions, “due regard” might be paid to the drug incorporation technique, which is considered particularly important for water insoluble or unstable substances. Nevertheless, there are only single approaches dedicated to the evaluation of the solvent casting method in the multilayer systems preparation [31,32,33,34]. It was found that a poorly explored area of multilayer films prepared by using the aforementioned technique might be a good point for further investigations. Due to the variety of possible polymer combinations and many factors determining the interpolymer complexation process, it gives wide perspectives for exploring.

**Table 2:** The utility of PEMs as drug delivery systems with regard to the polycation and polyanion used, model drugs, and potential applicability.

Polycation	Polyanion	Active Substance	Potential Applicability	Reference
Chitosan	Sodium alginate	Tamoxifen	Patches, injectable gel-like films	Criado-Gonzalez et al. [35]
	Hyaluronic acid/sodium hyaluronate	Doxorubicin hydrochloride, Fluorescein isothiocyanate, Ovalbumin	Anticancer rapidly-disintegrating films	Sun et al. [36]
	Casein sodium	Benzydamine hydrochloride	Buccal films	Pilicheva et al. [37]
	Heparin and dextran sulfate	Transforming GF β1, platelet-derived GF ββ, and insulin-like growth factor 1	Materials for tissue regeneration	Damanik et al. [38]
	Poly(γ-glutamic acid)	Interferon-γ	Systems for gastric cancer treatment	Cardoso et al. [39]
	β-cyclodextrin polymer	4-tert-butylbenzoic acid	Medical devices with antibiotics or antiseptic agents	Martin et al. [40]
		Gentamicin	Prevention of perioperative infections	Pérez-Anes et al. [41]
	Sodium salt of carboxymethyl cellulose	Fluorescein isothiocyanate, ovalbumin	Medical devices	Park et al. [42]
	Pectin/Xanthan gum/Karaya gum	Tenofovir	Vaginal films	Martín-Illana et al. [33]
Eudragit E^®^	Hypromellose acetate succinate	Diclofenac sodium	Colon-specific tablets	Jeganathan et al. [43]
Poly(4-vinylpyridine)	Sodium alginate	Ciprofloxacin hydrochloride	Transdermal systems	Alshhab et al. [31]

**Figure 3:**
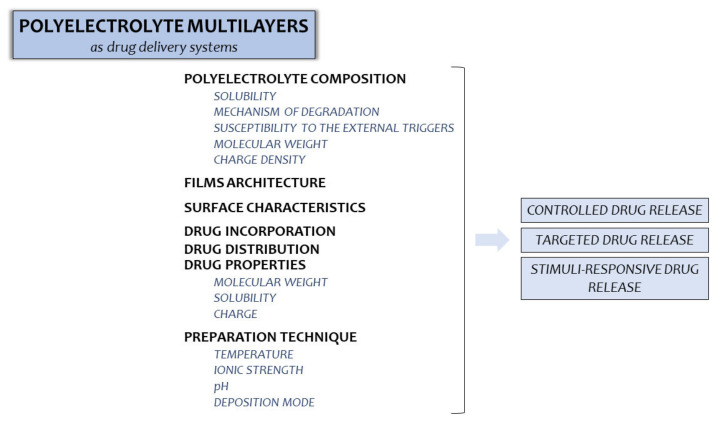
Factors which should be taken into consideration in PEMs designing as potential drug delivery materials.

The potential of PEMs in the development of drug delivery systems is presented in Figure 3; with simultaneous indication of factors being crucial for their optimization.

#### 2.1.1. PEMs as Drug Dosage Forms (Films, Patches, Tablets, etc.) for Skin and Mucosal Administration

A spray assisted Lbl technique was utilized in a study devoted to the low molecular weight chitosan/sodium alginate multilayer systems for local delivery of tamoxifen [35]. Thus, 0.25% (*w*/*v*) sodium alginate and 0.1% (*w*/*v*) chitosan solutions in acetate buffer adjusted to pH 3 for polyanion and pH 5 for polycation were alternately deposited on a glass slide in order to obtain a high ionization degree of the interacting polymers. Similarly, an ethanolic solution of tamoxifen was incorporated into the systems as an intermediate layer. Despite the film roughness, the authors noted the spraying deposition as a time-saving and easy to scale-up procedure, as compared to a traditional dipping Lbl technique. The in vitro dissolution test showed a prolonged in-time release of the active substance, up to 10 days, which could be easily modulated with the number of polymeric layers and the final film’s architecture.

Polycationic character of poly(4-vinylpyridine), which results from the presence of the pyridine ring, able to protonate under an acidic environment, was utilized for interpolymer complexation of sodium alginate and PEC-based multilayer film development [31]. They were intended as potential platforms for skin administration of ciprofloxacin hydrochloride. For that purpose, 1% (*w*/*v*) sodium alginate solution was casted on a Petri dish for solvent evaporation, then 1% (*w*/*v*) dispersion of poly(4-vinylpyridine) in 0.1 M HCl was poured onto the pre-jellified polyanion film for 20 min to enable PEC formation. After this time, the fabricated film was washed with distilled water and dried in the oven at 40 °C. Analogically, systems composed of three, four, or five interacting layers were obtained; however, no drying of the applied polyelectrolytes was performed, except for the basic sodium alginate layer. Drug loading was performed by 48 h of immersing the films in an antibiotic solution (12.5 ppm in 25 mL). Then, the drug-deprived solution was analyzed spectrophotometrically at 275 nm for quantitative determination of ciprofloxacin incorporated in the polymeric matrix. After 48h of immersion, about 74–89% of drug loading efficiency was obtained. Triple-layer films consisted of polyanion/polycation/polyanion and exhibited smooth (and more hydrophilic character) as compared to the others, which probably arose from the ability of free carboxylic acid groups for hydrogen bonding of water. The hydrophilic environment of the trilayer systems was also crucial for satisfactory entrapment efficiency of water-soluble ciprofloxacin hydrochloride and its low adsorption on the film surface. The authors noted that the sodium alginate layer was responsible for the increased swelling capacity of the systems. Considering pH-dependent solubility of poly(4-vinylpyridine), acidifying the release medium to pH 1.2 resulted in the enhanced disintegration of polycation layers and significantly more rapid antibiotic release.

To reduce limitations of a dip coating method in providing the sufficient drug loading, the effect of crosslinking agents: glutaraldehyde, sodium tripolyphosphate, and calcium chloride, on the pharmaceutical performance of multilayer buccal films with benzydamine hydrochloride, was evaluated [37]. Using poly(DL-lactide) as a negatively charged substrate, low molecular weight chitosan and anionic casein sodium salt were alternately deposited by a programmable slide stainer and then chemically cross-linked according to the designed pattern. Considering the attention given to the electrostatically interacting polymers, both pH and ionic strength of 1% (*w*/*v*) polyelectrolytes solutions were controlled. Chemical modification of the films resulted in the reduced swelling capacity and improved drug loading as a result of a more compact and dense structure formation. The swelling performance highly corresponded to the in vitro drug release manner since the addition of crosslinking agents delayed the benzydamine dissolution rate. Nevertheless, insufficient effects of glutaraldehyde, sodium tripolyphosphate, and calcium chloride on the prolonged delivery of benzydamine (~90–94% drug release after 2 h) was highlighted. Mucoadhesive properties of chitosan resulting from the ability to interact ionically with negatively charged sialic acid of mucin have been widely explored to date [44]. Buccal mucosa is an “application side” where high retentivity of an administered product is required and utilization of chitosan as a mucoadhesion enhancer is particularly helpful. The issue is how the interpolymer complexation of the polycation, by different polyanions, might affect its retention capacity, since PEC formation is related to a declined amount of amine groups able to interact with mucin. Considering the satisfactory mucoadhesive behavior of the films developed by Pilicheva et al. [37], the impact of both film architectures and ability to ionically interact with mucin should be equally undertaken. Accordingly, a decreased amount of free cationic amine groups of chitosan involved in the process of interpolymer complexation or the crosslinking process, and simultaneously crucial for sialic acid bonding, cannot be regarded as a factor limiting the systems retentivity. Combinations of chitosan with negatively charged hyaluronic acid, alginic, or tannic acid [36] were also evaluated. The oxidized silica wafers were used as substrates for better adhesion of the deposited chitosan. Systems were designed as potential platforms for controlled delivery of doxorubicin hydrochloride, as well as a (model) small molecule fluorescein isothiocyanate and macromolecular ovalbumin. Considering the effect of pH on the polyelectrolyte ionization degree, the authors modified pH of the polymers solutions to obtain morphologically-designed systems with either dense or porous structures, depending on the polyanion used. Accordingly, highly ionized chitosan chains were unable to penetrate deeply into the film structure, resulting in a dense layer formation. In contrast, chitosan/tannic acid systems, being mainly stabilized by hydrogen bonding, were characterized as macroporous and rough matrices. Morphology, the availability of carboxylic acid/amine groups, and charge of the incorporated active substances highly affected drug loading and release kinetics of the systems formed. It was observed that degradation of the film layers upon contact with the medium (PBS, 37 °C) was responsible for the initial drug release, while ionic interactions or H–H bond formations allowed for prolonged delivery of the incorporated agents. According to Das et al. [9], the decreased mobility of polyelectrolytes in an interfacial layer might have been accompanied by their low concentrations at the surface; thereby, increased disintegrations of external layers upon the erosion process could occur.

The utility of Lbl-assembled multilayer coatings, made from medium molecular weight chitosan and carboxymethyl cellulose sodium salt as materials for fluorescein isothiocyanate and ovalbumin, was also evaluated by Park et al. [42]. Contrary to the dominant preparation techniques [36,45], an anionic component was deposited as the first one onto the epoxy-modified silica wafers. The excessive amount of the interacting polyelectrolytes was removed by using deionized water, and the built-up multilayers were subsequently cross-linked with 1-ethyl-3-(3-(dimethylamino) propyl)-carbodiimide hydrochloride/N-hydroxysulfosuccinimide and glutaraldehyde 25% solution. The pH of the oppositely charged polymer solutions were adjusted to 4, since at this point, the highest ionization degree of the polyelectrolytes was noted [46,47]. Fourier transform infrared spectroscopy (FTIR) spectra showed both electrostatic and hydrogen bond formation. Additional crosslinking resulted in the porosity and roughness increase, likely as a result of polymeric chain reorganization and new bond formation. In case of a fluorescein isothiocyanate–low molecular weight substance, the ability of ionic interactions with amine groups of chitosan not being involved in PECs was indicated as the main mechanism responsible for the drug loading. Porosity of the cross-linked films, in turn, affected high entrapment efficiency of macromolecular ovalbumin.

Timur et al., developed films and freeze-dried wafers consisting of medium molecular weight chitosan and neutral hydroxypropyl methylcellulose (HPMC) [32]. Even though they could not be treated as typical PEMs, being the combination of oppositely charged polyelectrolytes, it is worth it to recall this scientific paper, since it refers to the solvent evaporation method as one of the PEM preparation techniques. The composites were formed as platforms for cefuroxime axetil with potential use in the therapy of oromucosal bacterial infections. While the films were developed by two-step deposition of the polymer solutions on Petri dishes, with the weight ratio of chitosan to HPMC being 1:1.25, the technology of the wafers included the stage of freezing at −20 °C, then a 24h lyophilization process at −80 °C [32].

The PEM preparation technique proposed by Martín-Illana et al., combined the solvent evaporation method with the stage of exerting a constant pressure (10 N for 5 min) in order to obtain trilayer films for vaginal delivery of tenofovir [33]. The authors tested the combinations of ethylcellulose responsible for controlled drug release and chitosan being the mucoadhesion enhancer with a polyanionic component: karaya gum, pectin, or xanthan gum. In relation to the observations made by these authors on hybrid (inorganic and organic) porous materials as promising composites for biomedical purposes [33], inorganic drug release modulators synthesized from triethoxysilane and polydimethylsiloxane were incorporated in the films. The attention given to the hydration dynamics of the developed films allowed observation of the correlation between increasing water penetration induced by an inorganic drug release regulator addition and more favorable hydrogen or ionic bond formations between chitosan and karaya gum. Simultaneously, the interpolymer complexation of both karaya and xanthan gum limited the water uptake capacity of the PEC-based matrices but the swelling degrees of the films with ethyl cellulose, chitosan, and pectin were slightly higher as compared to the bilayer systems deprived of chitosan. Films with pectin demonstrated the lowest mechanical strengths, which excluded them from potential applicability on the vaginal mucosa. Similar to the results obtained by Pilicheva et al. [37], it was observed that the chitosan layer was able to provide mucoadhesive effects after PEC formation. In addition, this polycation was responsible for the enhanced mechanical properties of the triple-layered composites. Although release kinetics of physically mixed tenofovir was faster from the polymeric matrix enriched with xanthan gum in comparison to karaya gum, the addition of an inorganic drug release resulted in a less cross-linked, low molecular weight chitosan/karaya gum complex formation, with the improved ionic mobility of the simulated vaginal fluid responsible for rapid drug diffusion. In contrary, no significant changes in the hydration dynamics of the films with xanthan gum were noted, despite the presence of tenofovir release regulators. In conclusion, the triple-layered composites with xanthan gum and karaya gum were recognized as promising drug carriers for tenofovir due to the high retention capacity provided by chitosan and the prolonged release of the antiviral agent from PEC matrices modified (or not) by drug release regulators.

#### 2.1.2. PEMs as Coatings for Colon-Specific Tablets

**Figure 4:**
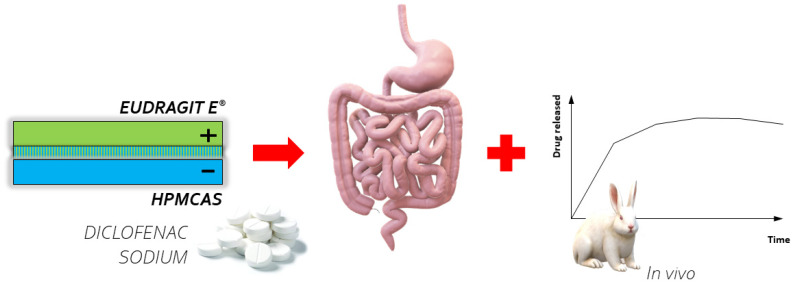
PEMs composed of cationic Eudragit E^®^ and anionic hypromellose acetate succinate (HPMCAS) as coatings for colon specific tablets.

The importance of multilayer systems in controlled drug delivery was highlighted in the study on the utilization of cationic Eudragit E^®^ and anionic hypromellose acetate succinate (HPMCAS) PEMs in bi- and triple-layer coatings of colon-specific tablets with diclofenac sodium, by Jeganathan et al. [43] (Figure 4). The cores of the tablets were coated in a perforated pan coater using a 10% (*w*/*v*) HPMCAS solution in acetone and a 10% Eudragit E^®^ solution in isopropyl alcohol. To determine the optimum polyanion/polycation ratio, turbidities of different polyelectrolytes mixtures were measured. Turbidity analysis is a simple and useful method to indicate the point of insoluble stoichiometric PEC formation [48,49,50]. Along with the development of pH-dependent PEM-based coatings to prevent the unwanted release of diclofenac sodium in the stomach, the addition of citric acid for better drug solubility was regarded as crucial for the intended drug release in the lower part of the gastrointestinal tract. In vivo studies in rabbits confirmed the delayed and prolonged delivery of diclofenac sodium.

#### 2.1.3. PEMs as Platforms for Biomolecules

Considering the high costs of bioactive molecules, such as growth factors (GFs), ensuring satisfactory drug loading is one of the most important aspects of successful GF-loaded platform development. Furthermore, their short half-lives and the risk of overdosing are the reasons that highly controlled drug delivery must be provided.

According to Kulkarni et al., layers of chitosan and negatively-charged epidermal GF (EGF) were successfully deposited onto polyurethane films by using the dipping Lbl technique; thereby, 5-day active substance delivery intended for wound therapy was achieved [51]. It might be assumed that electrostatic interactions between chitosan and EGF were crucial for the high encapsulation efficiency and over 90% bioactivity of EGF in the cell proliferation assay. Similar investigations were presented by Damanik et al., which aimed to evaluate Lbl assembly with regard to loading efficiency of various cationic GFs (transforming GF β1, platelet-derived GF ββ, and insulin-like growth factor 1) [38]. For this purpose, negatively charged sulfated polysaccharides—heparin and dextran sulfate—were used as protecting agents. Aside from the well-developed technological process of PEMs preparation, numerous factors, including pH, temperature, type of a substrate activation, time, or sequence of polyelectrolytes deposition were taken into consideration. In response to the assumptions of the study, selection of heparin, the slightly acidic environment (pH 5), and priming the substrate with oxygen gas plasma were responsible for the highest loading efficiencies for all investigated GFs. Functionalization of PEMs for sustained delivery of GFs was based on the careful selection of the polyelectrolytes creating multilayer composites. Kulkarni et al., emphasized the idea of mixing weak and strong polyelectrolytes as useful tools for the release mode optimization. Similar observations were made by Glinel et al., and Tan et al. [52,53]. In fact, weak polymers affect linearly growing systems while strong electrostatic interactions lead to an exponential mode of PEM increase, being responsible for prolonged drug release. Thus, 14-day bioactivity of the analyzed implants with GFs was accomplished, as confirmed in the in vitro assays by the enhanced fibroblast proliferation and myofibroblast differentiation.

Regarding the possibilities of PEMs in the delivery of various bioactive molecules, comprehensive investigations into chitosan/poly(γ-glutamic acid) PEC-based systems as platforms for interferon-γ (INF-γ) were also described, with special attention given to the assessment of their biological effects on macrophages activity [39]. Limited usability of INF-γ in anti-cancer therapies mainly results from high toxicity of the protein after systemic use. The necessity of the topical drug delivery system development led to several approaches focused on controlled delivery of INF-γ by using nano- and microparticles, liposomes, gels, or, according to Cardoso et al. [39], multilayer films. It should be noted that preparation techniques that eliminate the usage of organic solvents or temperature are highly recommended for bioactive molecules, considering their low stability against the aforementioned factors. Applicability of the multilayer systems in gastric cancer treatment was evaluated, based on INF-γ-induced transitions of pro-tumor macrophages to the tumor suppressor phenotype. Positively charged INF-γ did not hamper the interpolymer complexation process. The in vitro dissolution test performed at 37 °C, at pH 7.4 revealed significant differences in the drug release rate, depending on INF-γ content. While for the formulation with protein concentrations of 100 ng/mL, 90% of the dosage was released after 24 h, a five-fold increase in INF-γ content resulted in a significantly slower drug release (60% of dosage after 24 h). We should note that the incorporation of INF-γ inside chitosan and poly(γ-glutamic acid) layers had stronger effects on macrophages as compared to pure INF-γ.

Responding to wide opportunities that the spray-assisted Lbl method offered, in terms of coating geometrically diversified surfaces, Hsu et al. [54] developed a new technique of PEM fabrication using individual containment chambers for each aerosolized polymer or active substance solution. In this way, recycling the single components became possible and, therefore, the whole method was regarded as more feasible in an industrial scale production because of possible material recovery. Considering pKa values of oppositely charged polymers (cationic poly(β-aminoester) and anionic heparin) or the active molecule (lysozyme), pH was adjusted to 5 to provide sufficiently charged molecules for Lbl film build-up. Compared to the conventional spray Lbl technique, very similar release kinetics of lysozyme were obtained. Nevertheless, reduction of the spray time resulted in a more pronounced burst release effect [54].

The idea of embedding sensitive biomolecules inside the PEM structure is a well-known procedure with regard to peptide, protein, or small molecular delivery, as shown above. Nisin Z is an example of a natural antimicrobial peptide, in which its utility in the preparation of coatings in the pharmaceutical, food, and biomaterials sectors has been explored in the last few years [55,56,57]. Nevertheless, because of the purposed antimicrobial effect of the developed materials, we refer to investigations strictly devoted to PEMs as bacteria eliminating composites in the following section.

#### 2.1.4. PEMs as Coatings for Implants with Antimicrobial Activity

Researchers are drawn toward the design and development of medical devices covered with antimicrobial agents, since these devices can provide active substances in the affected areas without any additional invasive procedure [58]. In fact, postoperative and implant-related inflammation, as well as infectious diseases, remain serious clinical problems.

According to the investigations carried out by Pérez-Anes et al. [41], a negatively charged β-cyclodextrin-based polymer was selected as a multidrug reservoir while low molecular weight chitosan served as a barrier for controlled drug release. Cyclodextrins are widely known structures with amphiphilic nature resulting from the presence of hydrophilic exteriors and hydrophobic interiors, which enable reversible inclusion of water-insoluble therapeutic agents [26,59]. The PEMs were attached to the titanium platform by using polydopamine film, being a surface primer, improving polyelectrolyte anchorage to the underlaying substrate. The β-cyclodextrin-based polymer was synthetized by a polycondensation reaction between β-cyclodextrin and citric acid according to the previously optimized procedure [60]. Because of the biodegradability, biocompatibility, and polyanionic character, the polymer was chosen as an optimal component for chitosan, ensuring the sustained release of gentamicin—a broad-spectrum aminoglycoside antibiotic dedicated to perioperative infection treatments. Antimicrobial activity of the surfaces against *Staphylococcus aureus* was preserved for up to 6 days. By modulating the number of polyelectrolyte layers, the drug dissolution rate was easily modified.

In the study by Martin et al. [40], the poly(ethylene terephthalate) substrate was pre-treated, using the pad–dry–cure process, where the softener, crosslinking agent, catalyst, and other components were dried on the fabric prior to the crosslinking reaction taking place during the curing step [61], and then the substrate was utilized for immobilization of low molecular weight chitosan and the β-cyclodextrin polymer, as above. To evaluate the potential of PEMs as drug carriers, 4-tert-butylbenzoic acid, known for its capability of forming a stable inclusion complex with the polyanion, was selected as a model active substance. Similar to the previous investigations, the impact of ionic strength on the film thickness was described. High ionic force hampered the PEM growth and resulted in a thin and rough system preparation, most likely due to the transition in conformation of the interacting polymers upon ion concentration increase. At extremely high ion concentrations, the desorption of swollen polymeric layers was additionally observed. Drug loading and release behavior were noticeably correlated with the number of layers, nevertheless, differences between degradation and release mode suggested dominance of a diffusion mechanism above erosion in 4-tert-butylbenzoic acid delivery.

Both antibacterial and osteogenic coatings formed from cationic poly-L-lysine and anionic chondroitin sulfate A were developed by Roupie et al. [55]. While chondroitin sulfate A was responsible for bone cell stimulation, incorporation of nisin Z—a positively charged peptide obtained from *Lactococcus lactis* and active against Gram positive bacteria [62]—was evaluated with regard to antibacterial behavior against *S. aureus*. Glass coverslips pre-treated with poly(ethyleneimine) solution were utilized as substrates for Lbl growth. Besides formulations composed of six units of polycation and polyanion, the crosslinking effect of genipin on film mechanical performances and their resistance to enzymatic degradation and microenvironmental fluctuations was also assessed. The coating preparation was supported by Quartz Crystal Microbalance with Dissipation monitoring (QCM-D)—a technology measuring mass changes and viscoelastic properties of the surface-adhering layer. Decreasing roughness and stiffness of the films was most likely affected by the ingredient reorganization, with almost complete internal loading of nisin Z in the films matrices. Antibacterial properties of the antibiotics increased gradually, up to 48 h. After this time, no significant changes were noted. According to the LIVE/DEAD^®^ assay (two-color test to establish cell viability in a population based on plasma membrane integrity and esterase activity), PEMs did not express antiadhesive properties toward *S. aureus* culture and all surfaces were homogeneously covered with bacteria. In the study by Webber et al. [57], PEMs consisted of high molecular weight chitosan and the mixtures of κ- and λ-carrageenan were evaluated as potential delivery platforms of nisin Z. In comparison to the highly prolonged antimicrobial behavior of the films designed by Roupie et al., 6.5h activity against *S. aureus* and methicillin-resistant *S. aureus* were noted. Being “differently located” within the PEM structure’s active agent neither affected the surface properties (wettability, roughness) nor the therapeutic effects of the film. All samples were hydrophilic, with the contact angle in the range of 20–27°; nevertheless, the inclusion of nisin Z with hydrophobic residues across the N-terminal of the peptide was most likely correlated with the presence of gas bubbles inside the films. According to the antibacterial test, no activity of the placebo films related to the presence of antimicrobial chitosan was recorded. It might have been a consequence of limited number of amine groups of chitosan, being normally “responsible” in their free forms for antibacterial or antifungal behaviors [63]. Moreover, a relatively low concentration of chitosan solution utilized for the film preparation (100 ppm = 0.01%) might have been insufficient for bacteria reduction as well as a pH of 6 for the interacting components not adequate for a high ionization degree of chitosan’s amine groups.

### 2.2. Utilization of PEMs in Tissue Engineering

**Figure 5:**
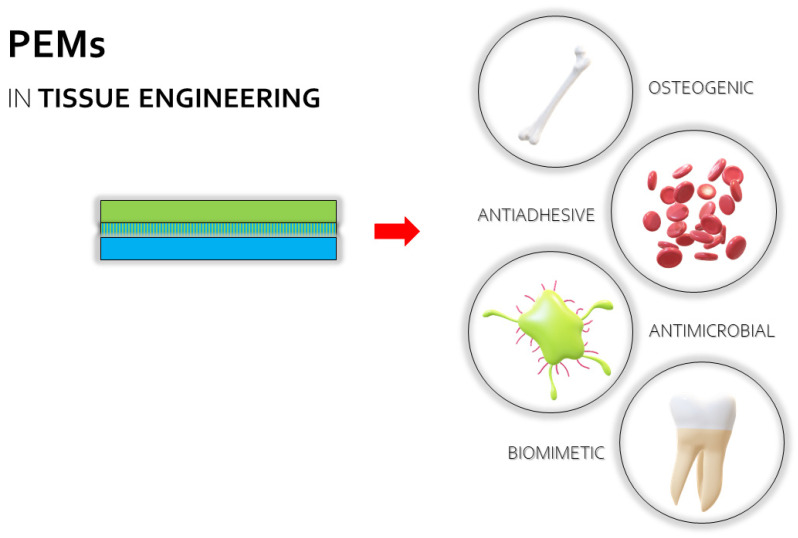
The possible utilization of PEMs in tissue engineering.

The great amount of scientific reports dedicated to the usability of PEMs in the fabrication of implantable materials leaves no doubt about the high potential of these structures in tissue engineering. The following investigations focus on different aspects of regenerative medicine or biomedical engineering (Figure 1), with special attention given to PEMs as potential antimicrobial coatings of implants and medical devices. Considering the widely known healing properties of chitosan and hyaluronic acid, the evaluation of their utilities in antimicrobial, regenerative, or biomimetic coating developments is the main subject of this section (Figure 5).

#### 2.2.1. Antimicrobial and Antiadhesive Coatings

Richert et al. [64] investigated the processing factors affecting growth of chitosan/sodium hyaluronate PEMs as promising antimicrobial coatings with potential use in tissue engineering. The polymer solutions were prepared using filtered saline solutions with different concentrations of NaCl, using an automatic dipping machine. The excess of the polycation/polyanion was removed by two-fold dipping in an NaCl solution used to prepare the polymer solutions. Better diffusion properties of low molecular weight chitosan cast at the surface of sodium hyaluronate, and then faster growth of the film, were noted. Furthermore, upon increasing the salt concentration (from 1 × 10^−4^ M to 0.15 M NaCl), more rapid build-up of the multilayer system was observed. The films obtained at high NaCl concentrations were chondrocyte and bacterial resistant, which might have been related to their uniform character and extremely smooth surface, limiting the mechanical support for pathogens. An 80% decline of *Escherichia coli* adhesion was noted after 30 min of the cell culture. Similarly, Hernandez-Montelongo et al., aimed to assess the antibacterial effects of the nanofilms composed of chitosan and sodium hyaluronate against *S. aureus* and *Pseudomonas aeruginosa*, which are regarded as the most common pathogens in nosocomial infections [65]. Properly prepared silicon wafers used as substrates were covered alternately with dipolymeric nanofilms by using the automatic dipping procedure. Both 1% (*w*/*v*) polymer solutions were prepared in 0.17 M NaCl and pH was adjusted to 2, 3, and 4.5 with 0.1 M HCl and/or 0.1 M NaOH in order to preserve the ionized form of cationic chitosan responsible for the antimicrobial properties of the system or its ability to interact ionically with sodium hyaluronate. Chitosan solution was also dispersed in 100 mM glacial acetic acid. The ionization degree values calculated for sodium hyaluronate showed the flat chain conformation of the completely ionized polymer at pH 4.5 as mainly responsible for the strong electrostatic interaction with chitosan, while loopy structure characteristics of the less charged sodium hyaluronate (mainly at pH 2) might have limited the PEC formation [66,67]. Thus, more intense polymer–polymer interactions resulted in the development of thinner and more rough films with decreased swelling ability as compared to these formulated at pH 2–3. Among all multilayer systems, the films prepared at pH 3 showed the highest antimicrobial effects against *S. aureus* during the 8 h test. It was explained by the largest amount of free positively charged amino groups on the film surface responsible for the antibacterial behavior. This correlates with the existing data, showing the polycationic nature of chitosan as the main mechanism determining the antibacterial effects of chitosan-based carriers. It is in fact assumed that electrostatic interactions between amino groups of the polymer and negatively charged cell surface or genetic material of the bacteria might be a reason for the polycation’s antibacterial behavior [65]. Considering the observations made by Richert et al., as well as the morphological differences noted for the analyzed films, the potential impact of the system roughness on their final antibacterial effects cannot be overlooked. Simultaneously, no significant colony reduction was observed for *P. aeruginosa*, what might be related to its morphological features, determining high microbial resistance.

Apart from the issues related to post-operative infections, tissue adhesion is one of the main problems associated with the healing process of damaged structures [68]. It is strictly related to the imbalance between the fibrin formation and the fibrinolysis process in the inflammation process, which often develops when the basal layer of the tissue is severely damaged. Antiadhesive materials, being physical, chemical, or the most popular biological barriers, are constantly developed to prevent post-operative tissue adhesion or adhesion connected with blood containing applications or cell sheet engineering. While physical barriers aim to physically separate the damaged structures from the surrounding healthy ones, chemical barriers are reservoirs of drugs, including fibroblast DNA suppressors, immunosuppressive, anticoagulation, fibrinolytic, or anticancer agents. Since high amounts of the aforementioned drugs are essential for antiadhesive effects, a great risk of potential adverse effects occurs. Accordingly, the development of coatings with controllable delivery of an active substance is highly demanded.

Experiments performed by Martins et al. [45] showed strong antiadhesive and bactericidal performances of chitosan/high methoxy pectin and chitosan/ι-carrageenan surfaces against *P. aeruginosa* and *S. aureus*. Consecutive depositions of the polymer solutions were performed on a glass surface subjected to the oxidation process to enhance adsorption of cationic chitosan. Moreover, 1 mg/mL polyelectrolytes dispersions were prepared using an acetic acid/acetate buffer solution adjusted to pH 5. Compared to chitosan/pectin multilayers, a combination of chitosan and ι-carrageenan resulted in less rough and more wettable material formation. The measurement of wettability allows to evaluate the hydrophilic/hydrophobic character of a surface and ensures its desired biological performance. A water contact angle above 90° is characteristic of hydrophobic surfaces while values below 90° describe systems with more hydrophilicity [69]. For multilayer systems developed by Martins et al., the values of this parameter oscillated around 25° confirmed the hydrophilic nature crucial for supporting the bone marrow stem cell adhesion, proliferation, and spreading. Cytocompatibility and strong antibacterial activity of the designed biomaterials revealed their potential applicability in tissue engineering with special emphasis on the orthopedic implant development.

#### 2.2.2. Regenerative Coatings

According to the outcomes obtained by Barroso et al. [70], 0.1% (*w*/*v*) low molecular hyaluronic acid and 0.1% (*w*/*v*) medium molecular weight chitosan were deposited onto a poly(ethylene terephthalate) surface with simultaneous removal of non-adsorbed polymer by the acetate buffer. Regarding the simultaneously-prepared hydrogels composed of precipitated PEC particles, separated after mixing the polyelectrolyte solutions at a weight ratio of 1:1, the multilayer systems were characterized by more intense polymer–polymer interactions observed in FTIR spectra. Furthermore, the authors noted poor swelling capacity and low repeatability of the obtained results, indicating inhomogeneous deposition of the polymers on poly(ethylene terephthalate) surface. Considering the hydrophobic character of the substrate, it could be concluded that its weak anchoring properties might have been responsible for the hampered adsorption of the polyelectrolytes on the surface and inhomogeneous film formation. Accordingly, prior priming of the hydrophobic surfaces, such as poly(ethylene terephthalate) and poly(tetrafluoroethylene) poly(ethylene), polycarbonate) using catecholamine polymers is therefore a common procedure in Lbl film technology. A differential scanning calorimetry analysis confirmed the polycation–polyanion interactions due to the variations in glass transition temperature values as compared to the thermograms recorded for single polymers [70]. Due to the reversible association–dissociation processes between oppositely charged groups of chitosan and hyaluronic acid, the multilayer systems were classified as self-healing materials with the ability to repair themselves after damage, which shows their potential utilization in biomedical sciences.

Apart from the aforementioned approaches toward chitosan/hyaluronic acid PEM-based material development, the potential of both polyelectrolytes in the technology of electrospun composites was also explored. Chitosan and hyaluronic acid were subjected to the needleless electrospinning technique and cytocompatibility of the produced nonwoven materials with mesenchymal stem cells (MSCs) was subsequently assessed [71]. Since MSCs are regarded as very important in regenerative medicine because of their trophic, multipotent, and immunomodulatory properties, the development of polymeric materials able to promote cell growth is needed. As both chitosan and hyaluronic acid are widely known for their healing and regenerative properties, their inclusion in electrospun biocompatible material was performed. Electrospinning is a technique that uses electric force to produce fiber from polymer solutions or melts. It combines two types of methods: electrospraying and conventional dry spinning of fibers for the nanostructures production. Apart from the monolayer chitosan or hyaluronic acid nonwovens and films, bilayer materials were also prepared [71]. While the monolayer systems were obtained by alternate depositions of the polymer solutions with the addition of polyethylene oxide as an electrospinning–promoting agent, the bilayer films were formed on a glass substrate by casting the polyanion onto a pre-jellified chitosan layer using a spinneret. The concentrations of chitosan and hyaluronic acid dispersions were 2% and 4%, respectively; however, no attention was drawn to the pH or ionic strength conditions. As compared to chitosan-based nonwovens, the bilayer composites performed significantly lower porosity and water uptake ability, which might have resulted from intense electrostatic interactions between chitosan and hyaluronic acid and, thus, dense and compact PEC formation. Furthermore, the authors emphasized that ionically interacting polyelectrolytes might have promoted polymorphic modifications of chitosan, which potentially reduced hydrophilicity of the whole system. The electrospun materials exhibited noticeably higher porous architecture as compared to the films, and their beneficial effects on cell viability and differentiation were confirmed in biocompatibility testing [71].

#### 2.2.3. Coatings for Coronary Stents

In a study by Nolte et al. [72], the cytotoxicity of chitosan/hyaluronic acid multilayers was assessed. According to the potential applicability of the systems as coatings for coronary stents, the effects of single polyelectrolytes, as well as the polyelectrolytes in combinations on the cell viability, were investigated. Even though it was shown that low rigidity of the films was responsible for the decreased cell proliferation rate, no cytotoxic effect was induced by the film components themselves, confirmed in the “reverse assay”, where the coated substrates were placed on top of the pre-cultured cells and were then observed. In the parallel experiments on the sulfonated polystyrene/polyallylamine hydrochloride systems, destructive influence of the highly charged synthetic polymers on the cell viability was highlighted. Accordingly, it can be concluded that the “stoichiometricity” of PECs might be regarded as a valid factor encouraging cell proliferation.

#### 2.2.4. Biomimetic Coatings

As compared to antiadhesive materials, biomimetic scaffolds for tissue engineering and regenerative medicine aim to mimic the physiological environment of tissues and organs by tissue/cell adhesive application in order to assist and accelerate their regeneration [73].

Regarding the biofunctionalization of medical devices—Rudt et al., focused on chitosan and hyaluronic acid as potential biomimetic coatings for biomedical purposes [74]. In response to their weak adhesiveness to human umbilical vein endothelial cells, the effect of graphene oxide incorporation on the material properties was investigated. Graphene oxide is an amphiphilic, high surface area, excipient rich in oxygen-containing functional groups, e.g., carbonyl, carboxyl, hydroxyl, or epoxide groups, and is known in pharmaceutical technology as a vehicle for gene or anti-cancer drugs [75,76]. Rudt et al., developed PEMs via alternate depositions of polycation, polyanion, and graphene oxide (0.5 mg/mL) dispersed in 250 mM NaCl with the pH adjusted to 5.5. As it is routinely performed, rinsing the excess of the polyelectrolyte prior to the following deposition step by using the dissolving medium, was carried out. Since the surface wettability was recognized as an important feature for cell adhesion, the effect of graphene oxide on the material hydrophilicity was measured. Due to the presence of hydrophilic functional groups, the excipient addition resulted in the decline of the water contact angle. Furthermore, the increased roughness and cellular adhesion were noted for graphene oxide-enriched coatings.

### 2.3. PEMs in Chemical Technology

Potential utilization of PEMs in the pervaporation separation of liquid mixtures has been widely investigated in recent years because of their high output and selectivity as compared to typically used cellulose acetate or polyvinyl alcohol membranes [77,78,79]. It is thought that polyelectrolytes with high charge densities (mainly synthetic polyanions) are responsible for good separating properties of the membranes.

Chitosan/λ-carrageenan films were prepared by Lbl deposition of an acetic acid solution of 1.5% (*w*/*w*) chitosan and 1.5% (*w*/*w*) λ-carrageenan solution in water with using a laboratory spinneret [78]. After gelation of the polyanion layer, chitosan was deposited and then films were dried at room temperature. For removing uncomplexed λ-carrageenan and chitosan, either water or 2% acetic acid were utilized, respectively. A similar investigation was carried out by Kononova et al. [79] for chitosan/sodium hyaluronate multilayer films prepared by using 2% (*w*/*w*) solutions of the polymers with different deposition orders. The ratio between polycation and polyanion charge densities was estimated at 1.6:1. According to the X-ray diffraction (XRD) technique, which provides detailed information about the crystallographic structure, chemical composition, and physical properties of the analyzed materials, the order of polyelectrolyte deposition profoundly influenced structuring of the polycomplex. The deposition of sodium hyaluronate on a jellified chitosan layer resulted in significant reorganization of the polycation chains. Furthermore, casting sodium hyaluronate on the chitosan surface resulted in dense and ordered PEC formation, while the reverse order of ingredient addition affected the loose structure of the interfacial layer. The thermal analysis revealed the structural variety in the film architecture. The layer of PEC was characterized by high thermal stability.

In the study performed by Petrova et al., polysaccharide films composed of a 2% solution of medium molecular weight chitosan and a 2% solution of polyanion, including hyaluronic acid, alginic acid, or a mixture of different types of carrageenans, were developed by using a spinneret [77]. Except for chitosan/carrageenan formulations—the polycation solution was casted as the first one. Chitosan/alginic acid films were characterized by the highest swelling abilities, most likely as a result of the polyanion block structure, which promoted formation of a porous PEC [80]. As the morphology analysis—by using scanning electron microscopy—showed, the microstructure of the polycomplexes layer was determined by the polyanion used. Furthermore, the transition of chitosan from hydrated to anhydrous polymorphic form, as a consequence of PEC formation, was observed by using XRD analysis.

## 3. Conclusions

Polyelectrolyte multilayers possess undeniable potential in tissue engineering. Apart from their wide usage in the technology of antiadhesive, regenerative, or biomimetic materials, with therapeutic effects coming from polymer multifunctionality, a large part of the system represents antimicrobial agent-loaded coatings for implantable structures. Layer-by-layer assembled composites (films or wafers) are also recognized as promising drug delivery platforms for strictly pharmaceutical purposes. As structurally diversified forms of dosages, they provide wide perspectives of multifunctional system development via the selection of polymer layers responsible for the desired application properties, e.g., mucoadhesive or modifying drug release effect. Moreover, controllable interpolymer complexation might be a valuable source of polyelectrolyte structures with unique characteristics when it comes to therapeutic or physicochemical behavior. Nevertheless, regarding the plethora of factors affecting polycation–polyanion interactions, careful adjustment of the technological procedure is crucial for successful multilayer formation. It is expected that deeper insight into the newest scientific approaches concentrated around technology and physicochemical characteristics of the polyelectrolyte multilayers by using advanced analyzing procedures is crucial for their wider utilization in the pharmaceutical technology. In our opinion, these systems might effectively serve as drug carriers intended for mucosal administration due to their simplicity, comfort of dosing, small size, and high stability. Concurrently, multicomponent and, thus, multifunctional characters of multilayer systems with extra mucoadhesive effects, modified drug release modes, controllable swelling, or disintegration processes, might be treated as particularly usable in mucosal drug delivery.

## Figures and Tables

**Table 1:** Physicochemical characteristics of the selected polyelectrolytes used in PEM preparation [3,25,26,27,28].

Polyelectrolytes	Physicochemical Characteristics
**Polycations**	
*Natural origin polycations*	
Chitosan	Linear polysaccharide composed of β-(1,4)-linked D-glucosamine and N-acetyl-D-glucosamine units. Product of chitin deacetylation derived from the exoskeleton of crustaceans, insect cuticles, or fungi. Available in a wide range of deacetylation degrees and molecular weights. The presence of amine groups determines its polycationic character. Poorly soluble at a pH above 6.5. Multifunctional performance covers antifungal, antibacterial, anti-inflammatory, and healing behaviors of the polymer.
*Synthetic polycations*	
Eudragit E	Cationic polymer based on (dimethylamino)ethyl methacrylate and other neutral methacrylic acid esters. Soluble at pH below 5. Available in the form of an organic solution (Eudragit E 12.5), granules (Eudragit E 100), or powder (Eudragit E PO). Commonly utilized as film coating for drug dosage forms.
Poly(4-vinylpyridine)	Hydrophobic polymer with polycationic character at pH below 4.7.
Poly-L-lysine	Synthetic amino acid with positively charged hydrophilic amino groups. Widely used as a coating agent to support cell adhesion by altering surface charge in culture.
**Polyanions**	
*Natural origin polyanions*	
Sodium alginate	Linear polysaccharide composed of 1,4-linked β-D-mannuronic acid and α-L-guluronic acid units with anionic carboxylic acid groups. Hydrophilic derivative of alginic acid derived from marine brown algae by alkali extraction. Slowly soluble in water, forming a viscous colloidal solution susceptible to concentration, pH, temperature, or the presence of metal ions; while at a pH below 3, alginic acid precipitates, a pH above 10 induces viscosity decrease. Widely used as a thickening, suspending and stabilizing agent.
Hyaluronic acid	Linear polysaccharide composed of α-(1,4)-D-glucuronic acid and β-(1,3)-N-acetyl-D-glucosamine units with carboxylic acid groups. Derived from animal tissues or produced via bacterial fermentation with genetically modified strains. One of the major constituents of the skin and extracellular tissues. Determines cell growth, migration, and differentiation. Characterized by significant moisture retention and beneficial viscoelasticity.
Heparin	Mucopolysaccharide consisted of sulfated D-glucosamine and D-glucuronic acid with sulfaminic bridges. Obtained from mucosal tissues, e.g., porcine intestines or bovine lungs. Highly acidic polymer. Used as an anticoagulant.
Chondroitin sulfate	Sulfated glycosaminoglycan composed of the units of β-(1,4)-D-glucuronic acid and β-(1,3)-N-acetyl-D-galactosamine. One of the components of the cartilage and extracellular matrix. Widely used in combination with glucosamine in the therapy of osteoarthritis. Regulates adhesion, proliferation, and differentiation of cells.
Carrageenans	Potassium, sodium, calcium, magnesium, or ammonium sulfate esters of galactose and 3,6-anhydrogalactose copolymers obtained from red seaweeds. There are three types of carrageenans: kappa (κ)-, iota (ι)-, and lambda (λ)-carrageenans, with different numbers and positions of negatively charged ester sulfate groups. Various susceptibilities to interpolymer complexation is noted (depending on the isomer type). The polymers are recommended for oropharyngeal and buccal drug dosage form development because of high mucoadhesion properties. Widely used as stabilizing and thickening agents (substitute of gelatin).
Tannic acid	Plant polyphenol mostly derived from Caesalpinia spinosa, Rhus semialata, R. coriaria, and Quercus infectoria. Mixture of polygalloyl glucoses or polygalloyl quinic acid esters. Contains no carboxyl groups; weakly acidic character results from the presence of phenolic hydroxyl groups. Hydroxyl groups determine high water-solubility. Used for the therapy of diarrhea, skin burns, and rectal disorders.
Poly(γ–glutamic acid)	Naturally occurring, water-soluble, biodegradable, and non-toxic poly amino acid. Produced by *Bacillus subtilis*, *B. amyloliquefaciens*, *B. megaterium*, and *B. licheniformis* in a fermentation process. Stimulates immune activity. Utilized in chemotherapeutic agent delivery.
Xanthan gum	High molecular weight polysaccharide composed of D-glucose, D-mannose, and D-glucuronic acid monomers obtained from *Xanthomonas campestris.* Easily modified due to the presence of carboxylic acid and hydroxyl groups. Viscosity and mucoadhesion enhancer; stabilizing and prolonged release agent in oral and topical products. Stable over a wide range of pHs and temperatures.
Karaya gum	Product obtained from the exudate of *Sterculia urens* with a rhamnogalacturonan-type partially-acetylated ramified structure; mainly composed of α-D-galacturonic acid and L-rhamnose units. Relatively stable at acidic pH and thermolabile. Adhesive, emulsifier, suspension, and tablet agent.
*Synthetic polyanions*	
Hypromellose acetate succinate	Mixture of acetic acid and mono-succinic acid esters of hydroxypropylmethyl cellulose, obtained by the esterification of hypromellose with acetic anhydride and succinic anhydride. Enteric polymer soluble at a pH above 5.5 due to the presence of carboxyl groups. Creates clear or turbid solutions in buffers with pH above 4.5. Controlled-release, enteric-coating, or solubility-enhancing agent. Easily subjected to hydrolysis upon exposure to moisture.
Carboxymethylcellulose sodium	Sodium salt of poly(carboxymethyl) ether of cellulose. Easily soluble in water; forming transparent, colloidal solutions. Incompatible with ethanol 96%, strongly acidic solutions (pH < 2), metal salts, or xanthan gum. Viscosity-enhancing agent for oral and topical products. Because of its beneficial bioadhesive and water-uptake properties, it is utilized for wound healing. Drug- and cyto-protective agent.
β-cyclodextrins	Represent a family of cyclic glucopyranose oligomers derived from hydrolyzed starch via enzymatic degradation with a lipophilic interior cavity and a hydrophilic exterior. Consist of seven α-(1,4)-linked glucopyranose units. Limited water-solubility (1:50 at 20 °C, 1:20 at 50 °C). Form inclusion complexes for oral administration, providing physicochemical stability of the drug or taste-masking effect.

## Data Availability

Not applicable.
